# Molecular docking analysis of pyrrole derivatives with the human epidermal growth factor receptor 2: Combating breast cancer

**DOI:** 10.6026/973206300211075

**Published:** 2025-05-31

**Authors:** Stephen Ilango, Basaralu Yadurappa Sathish Kumar, Girija K.

**Affiliations:** 1 Department of Pharmaceutical Chemistry, Mother Theresea Post Graduate and Research Institute of Health Sciences (Government of Puducherry Institution), Gorimedu, Puducherry - 605006, India; 2 Department of Biotechnology, JSS College, Ooty road, Mysore - 570025, India

**Keywords:** HER2 target, SR9009, molecular docking and molecular dynamics

## Abstract

Breast cancer is a heterogenous disease and one of the leading causes of malignancy-related death in women. Synthetic pyrrole derivative of SR9009 has cytotoxic activity and hence it is attention to document virtual screening of SR9009 against HER2 target protein of breast cancer. A molecular docking result shows that SR9009 has higher binding affinity towards HER2 targeted protein. Molecular Dynamics results shows that SR9009 has higher binding energy for HER2 + SR9009 complex found to be -158.436 +/- 11.495 kJ/mol compared to HER2 + Trastuzumab complex found to be -134.772 +/- 19.859 kJ/mol. Hence SR9009 is clinical candidate molecule for targeting HER2 based on Molecular Docking and Molecular Dynamics studies for anti-breast cancer activity.

## Background:

Breast cancer accounts for roughly one-third of all malignancies in women, 15% of mortality rate contributes of total number of diagnosed cases. Multiple interplay of genetic, environmental and lifestyle factors influence the global distribution of breast cancer [1]. According per World Health Organization [WHO], 2.3 million women diagnosed with breast cancer and 670000 deaths globally in 2022 [2]. International Agency for Research on cancer [IARC] part of WHO classified as shift work involves disruption of circadian rhythm probably carcinogenic factor to humans reported in 2007 [3]. Study of research in disease prevalence has shown link between breast cancer and core component of circadian rhythm [4]. Human Epidermal Growth factor receptor 2 [HER2] targeted patient positive early breast cancer treated with chemotherapy. About 15% to 20% of breast cancer patients are HER2 positive, characterized by HER2 protein overexpression on an immunohistochemistry assay [5]. HER2 is one of a family of four membrane tyrosine kinase and important in pathogenesis and progression of human breast cancer. 85% majority of breast cancer due to HER2 signalling pathway major role in cell proliferation [6]. Night shifts nurses have changes in in gene of HER2 higher risk for developing breast cancer [7]. SR9009 a synthetic pyrrole derivatives specific target of Reverb alpha which core component of circadian rhythm to treat several circadian disorders including sleep disorders and cancer [8]. SR9009 shows anti-small cell lung cancer effect through inhibition of autophagy [9]. Trastuzumab a HER2 targeted monoclonal antibody and specific targeted to HER2 protein expression for the treatment of breast cancer. Overexpression of HER2 receptor triggers the growth of factor signalling pathway and its major driver factor for breast cancer. It activated and bind to Juxtamembrane domain of HER2 in receptor binding leads to down level of HER2 expression in breast cancer [10]. Pertuzumab a recombinant monoclonal antibody act on extracellular dimerization domain II of HER2, it down regulated ligand - dependent HER2 hetrodimerization leads to inhibition of tumor cell growth [11]. Therefore, it is of interest to describe the molecular docking analysis of pyrrole derivatives with the human epidermal growth factor receptor 2 towards combating breast cancer.

## Methodology:

Recent years Molecular docking became essential part of in-silico for drug development and it serving of virtual modelling method which facilitates to predict binding affinity orientation of ligand and receptor, when both interact with one another to form stable complex [12]. Pre-docking processes like ligand preparation, protein preparation and homology modelling.

## Hardware, software and website:

Hardware was used in laptop with Intel® CoreTM i7-1255U RAM 16.0 GB @ 1.70 GHz, 64-bit operating system at Windows 11. Molecular docking software using MGL tools 1.5.7 were downloaded from [13]. To remove water molecule, particular chain of protein converted into pdb format using Pymol 2.5.4 were downloaded from [14]. 2D structure into 3D structure using Avogardo 1.2.0 version were downloaded [15]. Missing sequence of protein were filled using Modeller 10.5 software were downloaded [16]. Visualization of protein and ligand interaction performed using Chimera X were downloaded from [17]. Regarding Molecular dynamics Groningen Machine for chemical simulation (Gromacs) software version 2020.4 were used. Swiss ADME was used to determine ADME properties of ligands [18]. Chemical structure of ligands was downloaded from Pubchem database [19]. HER2 protein structure was downloaded from RCSB Protein Data Bank [PDB] [20]. Active site of protein predicted using PDBsum [21]. Missing loop of protein sequences were filled using emboss needle [22]. Yasara online web tool were used for energy minimization of modelled protein [23]. PROCHECK was used for protein verification using Ramachandran plot analysis in PDBsum website was mentioned above. Regarding toxicity prediction, OSRIS property explorer open-source program was downloaded from [24]. Molecular dynamics study Ligand topology achieved using Automated Topology Builder [ATB] web server [25].

## Evaluation of ADME, physiochemical properties, drug likeness prediction and toxicity assessment:

ADME evaluation, Physiochemical analysis and drug-likeness assessment were conducted using Swiss-ADME web tool has been developed by Swiss Institute of Bioinformatics. Drug likeness screening was based on the criteria established by Lipinski [26], Ghose [27], Veber [28], Egan [29] and Muegge [30] rules of 5 screening. Bioavailability of ligands predicted using Abbot Bioavailability score. Toxicity profile of ligands predicted using OSRIS property explorer using colour code toxicity will be in red and safest in green colour for mutagenic, tumorigenic, irritant and reproductive effective.

## Ligand preparation:

The ligand molecules SR9009, Trastuzumab and Pertuzumab were downloaded from the PubChem database in 3D Standard Data Format (SDF) and subsequently converted to Protein Data Bank (PDB) format using PyMOL. SR9009 and Trastuzumab, has been downloaded 3D structure. SR9009 has been taken as test and trastuzumab, Pertuzumab consider as standard. Once the test ligand SR9009 obeys the Lipinski rule of 5 and Toxicity assessment afterward plan to perform docking analysis. Conversion from 2D to 3D structure was performed using Avogadro software [31]. Regarding Pertuzumab 2D structure has been downloaded and converted into 3D structure using Avogadro software.

## Protein preparation:

The 3D structure of the Human Epidermal Growth Factor Receptor 2 (HER2) protein, which plays a vital role in breast cancer, was obtained from the RCSB Protein Data Bank (PDB ID: 3POZ) in .pdb format. The selected protein corresponds to Homo sapiens and is relevant to human cellular systems. Targeted HER2 protein X-ray diffraction resolution should not more than 3.0 Å, which is 1.50 Å has been used. Ligand group, water molecules and hetero groups were removed from protein structure using Pymol [32].

## Homology modelling:

Missing amino acids of HER2 protein were constructed by homology modelling using MODELLER (Version -10.5). HER2 sequence was downloaded in Fasta sequence text format in RCSB PDB database considered as template. Gap sequence of HER2 protein were identified in Pymol and gap sequence aligned them with EMBOSS needle [33]. Energy minimization was done using Yasara online web tool. Modelled structure of HER2 protein was checked with PROCHECK to check quality of protein using Ramachandran plot. Protein have more than 90 percent and G-Factors has more than -0.5 value suggest that good quality of the protein recommend for Molecular analysis [34].

## Molecular docking:

Docking analysis was performed using Autodock tools software version 1.5.7 version. PDBsum database were used for Active site was prediction [35]. Hydrogen atom and kollman united atomic charges were added to the protein. Charged file of pdbqt of protein and ligand were prepared [36]. Grid map was set to 80x80x80 points with grid spacing of 0.375 Å. Docking parameter file (Dpf) and Grid parameter file (Gpf) were created to run the autodock and autogrid application. Genetic Algorithm (GA), 25 runs will be made to get docking configuration. Lowest binding score and hydrogen bond formation usually taken as the best docking score and visualized through Chimera X software [37].

## Molecular dynamics simulation analysis:

Groningen Molecular Simulation package (GROMACS) 2020.2 versions were used for Molecular Dynamics (MD) study. ATB server added heavy atoms using Ligand topology. By using steepest decent algorithm, prepared system was first vacuum minimized for 1500 steps. Solvated structures in cubic periodic box were assessed using Simple point charge water model (SPCE) 38]. Parameters were used for GROMACS 2020.4 software package including Root mean square deviation (RMSD), root mean square fluctuations (RMSF), Solvent accessible surface area (SASA), radius of gyration (RG), Principal component analysis (PCA), Hydrogen bonding (H-Bond), Free energy landscape (FELs) and Molecular Mechanics Poisson-Boltzmann surface area (MM-PBSA) were used to understand the binding energy with targeted protein over 100ns simulation time. To estimate the binding free energy GROMACS utility g_mmpbsa were employed [39].

## Results and Discussion:

ADME properties of ligands were shown in (Table 1) predicted that gastrointestinal absorption were high values in SR9009, Pertuzumab and low in Trastuzumab. Only Pertuzumab has BBB permeability and SR9009, Trastuzumab has no BBB permeability. No P-glycoprotein substrate for all the ligands. SR9009 shows that most of the cytochrome P450 isoenzymes were inhibited but only CYP1A2 isoenzyme was not inhibited. Trastuzumab has all cytochrome P450 isoenzymes were not inhibited and Pertuzumab only CYP2D6 isoenzyme were inhibited. Physiochemical properties and lipophilicity of ligands were predicted and shown in (Table 2) indicate that Hydrogen bond donar should be less than 5 but Trastuzumab greater than 5 which is 6 shows violation and SR9009, Pertuzumab values were smaller than 5 as per Lipinski rule of 5. For Topological Surface Area (Å) values show that for Transtuzumab 185.53 (Å) which is greater than 140 (Å) shows violation and for SR9009, Pertuzumab shows 106.84, 41.49 which is less than 140 (Å) as per Lipinski rule of 5. Water solubility predicted that trastuzumab were shown soluble when compared SR9009 and Pertuzumab show that moderately soluble. Molecular weight of the all ligands was less than 500. Lipophilicity shows that SR9009 (3.45), Trastuzumab (-2.64) and Pertuzumab (3.13). Toxicity profile of ligands, red colour shows in Pertuzumab was found to be mutagenic and green colour shows for both SR9009 and transtuzumab has been found that no toxicological features that have been obtained from OSRIS predictions. Overall ligands SR9009 and Pertuzumab obey the Lipinski rule of 5 but Trastuzumab shows the violations. Even though we take Transtumab as a standard ligand for docking analysis owing to it available as standard treatment drug for breast cancer patients. Active site predicted using PDBsum Lys 913 & Arg 803 was used for molecular docking analysis for HER2 targeted protein. Breast cancer target HER2 were docked with Trastuzumab and Pertuzumab, results reveal that SR9009 shows higher binding affinity (-8.81 kcal/mole) compared to trastuzumab (-6.8 kcal/mole) and Pertuzumab (-6.3 kcal/mole). Interaction of SR9009 with HER2 targeted protein and Hydrogen bond formations of SR9009 with HER2 target protein such as Asn147 & Lys184 were visualized shown in (Figure 1A and 1B see PDF). SR9009 predicted good binding affinity in breast cancer target HER2 when compared to Trastuzumab and Pertuzumab through virtual docking analysis. The study further extended to MD simulation study, SR9009 chosen as test system and among two standard ligands such as Trastuzumab and Pertuzumab among 2 standards, Trastuzumab were chosen standard system for MD simulation study based on docking score. MD simulation 100ns used to predict the stability of the HER2 target protein and SR9009 complex. To predict the precise result, 50ns with dt 1000 frames were run. MD simulation results predicted that 3 complex system APO (HER2 only) in black colour, Drug (SR9009 + HER2) in brown colour as Test and (Trastuzumab + HER2) in blue colour as standard system.

## Root mean square deviation:

During 10ns, all the equilibrium rise and remain stability for further remaining 90ns. After 100ns simulation period, RMSD reveals that HER2 only, SR9009 + HER2 and Trastuzumab + HER2 include 0.25 ± 0.02nm, 0.28 ± 0.04nm and 0.30 ± 0.04nm. By comparing 3 systems, SR9009 + HER2 complex and HER2 only has shown lower value reveals more stability compared to Trastuzumab + HER2 complex. At 60 to 80ns, Trastuzumab + HER2 complex shown maximum peak with 0.36nm when compared to both HER2 only and SR9009 + HER2 complex system were shown in (Figure 2 see PDF) after that remain stable for further simulation period.

## Root Mean square fluctuation:

To measure the fluctuation of 3 systems during 100ns simulation of period. Average value of HER2 only, SR9009 + HER2 and Trastuzumab + HER2 results reveal that 0.12 ± 0.09nm, 0.13 ± 0.13nm and 0.13 ± 0.14nm. Among that all 3 values similar and remain less fluctuation for all 3 system Between 150 to 200 residues slightly peak increased have higher fluctuation to 0.45nm after than remain stable for further simulation of period were shown in (Figure 3 see PDF).

## Radius of gyration:

To assess the dynamic compactness for 3 complex system HER2 only, SR9009 + HER2 and Trastuzumab + HER2. During 100ns simulation, Average values are predicted for HER2 only, SR9009 + HER2 and Trastuzumab + HER2 indicate that 2.07 ± 0.01nm, 2.04 ± 0.01nm and 2.03± 0.01 nm. By comparing 3 complex system, SR9009 + HER2, SR9009 + Trastuzumab shows lower value remain more compactness during 100ns simulation compared to HER2 only were shown in (Figure 4 see PDF).

## Solvent accessible surface area (SASA):

To determine the accessibility of HER2 protein molecule in solvent environment. Average values indicate for HER2 only, SR9009+HER2 and Trastuzumab + HER2 reveals that 168.64 ± 3.31nm, 165.88±4.81nm and 167.17 ± 5.00nm. By comparing these 3 values, SR9009 + HER2 complex system lower value indicate that decrease in surface area when interaction with protein and ligand. At the range of 170 - 180 nm^2^ all 3 system HER2 only, SR9009 + HER2 and Trastuzumab + HER2 were maintained were shown in (Figure 5 see PDF).

## Intra and inter hydrogen bond:

Over 100ns simulation period time dependent behaviour of Intra and Inter hydrogen bond were revealed for HER2 only, SR9009 + HER2 and Trastuzumab + HER2 for 3 system complexes were Intra HB shown in (Figure 6A see PDF) and Inter HB shown in (Figure 6B see PDF). Average value indicates that in time-dependent behaviour of Intra Hydrogen Bond for HER2 only [216.94 ± 9.16 nm], SR9009 + HER2 [222.80 ± 8.75 nm] and Doxorubicin + HER2 [223.43 ± 8.30 nm] reveals that 3 system significant for cable of Hydrogen bond formation. Regarding Inter-Hydrogen bonds were formed during simulation period and maintained by 1 to 6 hydrogen bonds for HER2 + SR9009 complex and 1 to 7 hydrogen bonds for HER2 + Trastuzumab complex. At last, both intra and inter-hydrogen bond were formed for both SR9009 and Trastuzumab with HER2 target protein, Hydrogen bond plays a vital role in respect to the stabilization of protein-ligand complex. SR9009 will be potential candidate against HER2 target.

## Principle component analysis (PCA):

To determine the collective movement in HER2 only, SR9009 + HER2 and Trastuzumab + HER2. Initial few eigenvectors play a crucial role in global motion of protein molecule. To assess the conformational dynamics of HER2 only and SR9009 + HER2 and Trastuzumab + HER2 during 100ns simulation period were shown in (Figure 7 see PDF) Time evolution of PCA for 3 system complexes, plot demonstrates that lower movement observed in SR9009 + HER2 and Trastuzumab + HER2 it did not significantly alter the target conformation and supporting the stability of the complex.

## Free energy landscape (FELs):

Global motion of the protein with the essential information for Free energy landscape. Analysis of FELs is common approach to investigate overall stability and folding mechanism of protein. Here we generate FELs plot for PC1 and PC2 were shown in (Figure 8 see PDF), blue colour area represents more stable protein conformation with lower energy. At 100ns simulation period, plot predicts range from 0 to 16 KJ/mol for HER2 + SR9009 and 0 to 14 KJ/mol for HER2 + Trastuzumab. SR9009 and Trastuzumab complex display a single global minimum confirmed large local basin. HER2 + SR9009 and HER2 + Trastuzumab did not cause any significant change in target structure compared to HER2 only protein.

## Molecular mechanics / poisson - boltzman surface area (MM-PBSA):

Overall energy determined by the utility tool of g_mmpbsa for GROMACS. Vanderwall energy predicted that HER2 + SR9009 complex shows that -262.401 +/- 26.331 KJ/mol were higher value compared to HER2 + Trastuzumab were -211.755 +/- 14.534 KJ/mol. Electrostatic energy shows that -99.502 +/- 47.477 KJ/mol for HER2 + SR9009 which has higher value compared to HER2 + Trastuzumab were -24.981 +/- 11.250 KJ/mol. Polar solvation energy shows that 276.014 +/- 73.208 KJ/mol for HER2 + SR9009 complex were greater value compared to HER2 + Trastuzumab complex were shown 123.355 +/- 16.859 KJ/mol. Binding energy shows that for HER2 + SR9009 complex were -158.436 +/- 11.495 KJ/mol were greater value compared to HER2 + Trastuzumab were -134.772 +/- 19.859 KJ/mol were shown in (Table 3). Overall MD simulation results predicted that HER2 + SR9009 complex indicate the stronger system stability and found tightly bind to HER2 protein compared to HER2 + Trastuzumab over 100ns simulation period. It will be considered as SR9009 may have clinical molecule due to greater affinity towards HER2 protein based on results.

## Conclusion:

Molecular docking and dynamics show that SR9009 has strong binding affinity to HER2 targeted protein, supporting further *In-vitro* and *In-vivo* validation.

## Figures and Tables

**Table 1:** ADME & Drug likeness of SR9009, Trastuzumab and Pertuzumab

**Properties**	**SR9009**	**trastuzumab**	**Pertuzumab**
**ADME**			
GI absorption	High	Low	High
BBB permeability	No	No	Yes
Pgp substrate	No	No	No
CYP1A2 inhibitor	No	No	No
CYP2C19 inhibitor	Yes	No	No
CYP2C9 inhibitor	Yes	No	No
CYP2D6 inhibitor	Yes	No	Yes
CYP3A4 inhibitor	Yes	No	No
Log Kp [cm/s]	-5.84	-10.31	-5.66
**DRUG LIKENESS**			
Lipinski violation	0	2	0
Ghose violation	0	1	0
Veber violation	0	1	0
Egan violation	0	1	0
Muegge violation	0	3	0
Bioavailability score	0.55	0.17	0.55

**Table 2:** Physiochemical and Lipophilicity of SR9009, Trastuzumab & Pertuzumab

**Properties**	**SR9009**	**Trastuzumab**	**Pertuzumab**
**Physiochemical Properties**			
Molecular Formula	C20H24CIN3O4S	C10H14N6O5	C17H27NO2
Molecular weight	437.94	298.26 g/mol	277.4
Hydrogen Bond Donar	2	6	2
Hydrogen Bond acceptor	5	7	3
Rotatable Bond	10	2	6
Molar Refractivity	120.13	69.9	83.33
Water solubility Log S [ESOL]	Moderately soluble	Soluble	Moderately soluble
Topological Surface area [A]	106.84	185.53	41.49
**Lipophilicity**			
TPSA	106.84	185.53	41.49
iLOGP	3.62	-0.93	3.48
XLOGP3	4.41	-3.08	3.28
WLOGP	4.11	-3.41	2.61
MLOGP	2.44	-2.85	2.42
SILICOS-IT Log P	2.66	-2.92	3.87
Consensus Log P	3.45	-2.64	3.13

**Table 3:** Molecular Mechanics/Poisson-Boltzmann surface binding energy for HER2 + SR9009 and HER2 + Trastuzumab

**System complex**	**van der Waal energy**	**Electrostatic energy**	**Polar solvation energy**	**Binding energy**
HER2-SR9009	-262.401 +/- 26.331 kJ/mol	-99.502 +/- 47.447 kJ/mol	276.014 +/- 73.208 kJ/mol	-158.436 +/- 11.495 kJ/mol
HER2-Trastuzumab	-211.755 +/- 14.534 kJ/mol	-24.981 +/- 11.250 kJ/mol	123.355 +/- 16.859 kJ/mol	-134.772 +/- 19.859 kJ/mol

## References

[1] Xiong X (2025). Signal Transduct Target Ther..

[2] Arnold M (2022). Breast..

[3] Hansen J, Pedersen JE (2024). J Natl Cancer Cent..

[4] Lin H, Farkas M.E (2018). Front Endocrinol (Lausanne)..

[5] Chen E (2025). Cancer Med..

[6] Gutierrez C, Schiff R (2011). Arch Pathol Lab Med..

[7] Li X (2025). Cancer Cell Int..

[8] Wagner P.M (2019). ASN Neuro..

[9] Shen W (2020). Theranostics..

[10] Gajria D, Chandarlapaty S (2011). Expert Rev Anticancer Ther..

[11] Ishii K (2019). Core Evid..

[12] Agu P.C (2023). Sci Rep..

[13] https://ccsb.scripps.edu/mgltools/downloads/.

[14] https://pymol.org/edu/.

[15] https://avogadro.cc/.

[16] https://salilab.org/modeller/download_installation.html.

[17] https://www.cgl.ucsf.edu/chimerax/download.html.

[18] http://www.swissadme.ch/.

[19] https://pubchem.ncbi.nlm.nih.gov/.

[20] http://www.rcsb.org/pdb/.

[21] https://www.ebi.ac.uk/thornton-srv/databases/pdbsum/.

[22] https://www.ebi.ac.uk/jdispatcher/psa/emboss_needle.

[23] https://www.yasara.org/minimizationserver.htm.

[24] https://www.organic-chemistry.org/prog/peo/.

[25] https://atb.uq.edu.au/.

[26] Lipinski C.A (2001). Adv Drug Deliv Rev..

[27] Ghose A.K (1999). J Comb Chem..

[28] Veber D.F (2002). J Med Chem..

[29] Egan W.J (2000). J Med Chem..

[30] Muegge I (2001). J Med Chem..

[31] Rajendran P (2023). J Compos Sci..

[32] Hu B, Lill M.A (2014). J Comput Chem..

[33] Chamata Y (2020). Int J Mol Sci..

[34] Wlodawer A (2017). Methods Mol Biol..

[35] Liao J (2022). Molecules..

[36] Ravichandran S (2022). Front cell Infect Microbiol..

[37] Pettersen E.F (2021). Protein Sci..

[38] Gangadharappa B.S (2020). J Biomol Struct Dyn..

[39] Kumari R (2014). J Chem INF Model..

